# Nonequilibrium Temperature: An Approach from Irreversibility

**DOI:** 10.3390/ma14082004

**Published:** 2021-04-16

**Authors:** Umberto Lucia, Giulia Grisolia

**Affiliations:** Dipartimento Energia “Galileo Ferraris”, Corso Duca degli Abruzzi 24, 10129 Torino, Italy; giulia.grisolia@polito.it

**Keywords:** irreversibility, nonequilibrium temperature, Gouy–Stodola theorem, Carnot theorem

## Abstract

Nonequilibrium temperature is a topic of research with continuously growing interest because of recent improvements in and applications of nonequilibrium thermodynamics, with particular regard to information theory, kinetic theory, nonequilibrium molecular dynamics, superfluids, radiative systems, etc. All studies on nonequilibrium temperature have pointed out that the definition of nonequilibrium temperature must be related to different aspects of the system, to the energy of the system, and to the energy fluxes between the system and its environment. In this paper, we introduce a definition of nonequilibrium temperature based on the Gouy–Stodola and Carnot theorems in order to satisfy all these theoretical requirements. The result obtained links nonequilibrium temperature to the electromagnetic outflow, generated by irreversibility during microscopic interaction in the system; to the environmental temperature; to the mean energy; and to the geometrical and physical characteristics of the system.

## 1. Introduction

Temperature represents a fundamental quantity in thermodynamics and statistical mechanics [1,2].

Recently, thermodynamics and statistical mechanics, both of small systems and of nonequilibrium systems, are increasing their roles in engineering and the sciences due to the theoretical and technological interest in micro- and nano-physics [3]. In this context, a new approach to the definition of temperature is required in order to avoid the conceptual difficulties from the classical definition, related to a macroscopic system [4].

In the history of physics, the first coherent approach to the definition of empirical temperature [5] originated in the 16th century, when Galileo began his studies on the design of the first thermometer [6], and then was improved by E. Torricelli and V. Viviani [7]. In relation to establishing and measuring temperature, a fundamental role is played by the zero law of termodynamics, which states that, if two systems are each in thermal equilibrium with a third, then they are in thermal equilibrium with each other [1,8]. By using this law, together with the Carnot’s theorem [1,2,9], the absolute temperature and its measure were introduced and the equivalence with the empirical temperature was shown [1,2].

After Daniel Bernoulli introduced the model of point-like particles for the ideal gas [10,11], Clausius proved that temperature is proportional to the mean kinetic energy [8,10,11,12]. After this first attempt at microscopic analysis, statistical mechanics was studied by Boltzmann [13], particularly with regard to the following [4]:

- The probabilistic interpretation of physical quantities;
- The link between the macroscopic and the microscopic worlds.

Consequently, a mechanical definition of temperature, *T* (K), was introduced in classical thermodynamics [14]:

(1) $$\frac{1}{T}={\left(\frac{\partial S}{\partial E}\right)}_{\mathrm{eq}}$$

where *S* is the entropy (J K${}^{-1}$) and *E* is the total energy (J) of the system considered.

Starting from these results, Einstein [15,16] proved that the fluctuations in physical observable quantities can be described in terms of macroscopic equilibrium thermodynamic functions, such that the energy of a system *E*, with internal energy *U*, results in the following:

(2) $$\langle E^{2}\rangle -{\langle E\rangle }^{2}=C_{v}\;k_{B}\;T$$

where $C_{v}$ is the heat capacity at constant volume (J K${}^{-1}$), proportional to the number, *N*, of particles in the system; $k_{B}=1.380649\times 10^{-23}$ J K${}^{-1}$ is the Boltzmann constants; and *T* is the temperature (K). Moreover, Einstein pointed out that fluctuations have a key role in statistical mechanics [4], and he showed the first example of the fluctuation-dissipation theorem by linking the diffusion coefficient to the mobility in Brownian motion. Recently, statistical mechanics of small systems [17] has grown important in theoretical and technological developments of micro- and nano-physics. In this context, fluctuations cannot be neglected, highlighting the relevance of the Boltzmann and Einstein results.

In relation to temperature, the Einstein result has been the subject of two controversial approaches [4]:

- A thermodynamic school states that it is possible to use the Einstein fluctuation theorem also for temperature [18,19].
- Another thermodynamic school states that, in the canonical ensemble used to describe the behavior of a system in contact with a thermal reservoir, the temperature is a parameter, and thus, it cannot fluctuate [20,21].

A response to this thermodynamic dichotomy was proposed by Mandelbrot using the estimation theory [22], obtaining the following relation:

(3) $$\langle {\left(\delta T\right)}^{2}\rangle =\frac{k_{B}}{C_{v}}\;T^{2}$$

which, considering the Equation (2), becomes

(4) $$\langle {\left(\delta T\right)}^{2}\rangle \;\langle {\left(\delta E\right)}^{2}\rangle ={\left(k_{B}T^{2}\right)}^{2}$$

usually considered the statistical thermodynamic analogy of the Heisenberg principle in quantum mechanics.

In our opinion, this result is valid without any doubt if we relate the quantity ${\langle {\left(\delta T\right)}^{2}\rangle }^{1/2}$ to the experimental measurement of temperature, as the uncertainty of the experimental result.

In this context, we must consider the fluctuation theorem. It was first introduced and verified using computer simulations in 1993 [23] but was first analytically proven in 1994 [24]. The first experimental proof of its validity was obtained in 2002 [25]. The fluctuation theorem deals with the relative probability that the entropy of a system that is currently not in thermodynamic equilibrium will increase or decrease over a given amount of time. This theorem concerns the probability distribution of the time-averaged entropy variation due to irreversibility, $S_{g}$. Indeed, it states that, in systems out of equilibrium, over a finite time interval τ, the ratio between the probability of $S_{g}=\Sigma$ and the probability of its opposite value, $S_{g}=-\Sigma$, is exponential in Στ, meaning that, as the time or system size increases, the probability of observing a value of the entropy production opposite to its value and obtained by the second law of thermodynamics decreases exponentially [26]. The validity of the fluctuation theorem has been proven in nonequilibrium statistical mechanics for any system also far from equilibrium. The fluctuation theorem refers to the dissipation function [26], and for this, it can be useful in our approach, based on the Gouy–Stodola theorem [27].

Therefore, the theoretical results on fluctuations [28,29] has led to the introduction of entropy production Σ as follows:

(5) $$\Sigma =k_{B}\;\ln\left(\frac{p(S_{g}=\Sigma )}{p(S_{g}=-\Sigma )}\right)\geq 0$$

where *p* is the density distribution of Σ, Σ=∫σdt, and $\sigma =\lim_{t\to 0}[k_{B}\;\ln$$\left(p\left(S_{g}=\Sigma \right)/p\left(S_{g}=-\Sigma \right)\right)]/t$ is the so-called phase-space compression factor [23,24,30]: In the original formulation [30], the quantity of the phase space compression factor is established under general conditions in steady states and it is related to the dissipated heat: the validity of (5) with σ equal to the rate of heat production has been experimentally verified [25,31]. Additionally, this result is the subject of many discussions on its interpretation, but from a phenomenological point of view, it can be interpreted as the result of the heat exchanged between the system and the environment. However, some comments on the macroscopic approach to classical thermodynamics must be developed. Indeed, in 1803, Lazare Carnot studied the efficiency of pulleys and inclined planes [32], finding analytical evidence of the conservation of mechanical energy. Then, in 1824, his son, Nicolas Léonard Sadi Carnot, developed a general approach to thermal engine by introducing its reference model and by analysing its maximum efficiency. He highlighted that the efficiency of a thermal engine always depends on the high and low working temperatures [9], with a value less than 1. Therefore, he first highlighted the fundamental role of environmental temperature in the inefficiency of any engine, process, or transformation [33,34,35]. However, real systems, contrary to the Carnot engine, are finite-size devices, operate in finite-time, experience dissipation and friction, and have efficiencies lower than the Carnot engine [12,14,27,36,37,38,39,40,41,42,43,44,45,46,47,48,49,50,51]. In the history of thermodynamics, a French physicist, Louis Georges Gouy (1854–1926) [52], and a Slovak engineer and physicist, Aurel Boleslav Stodola (1859–1942) [53,54], were crucial in developing a new theory for evaluating irreversibility. Indeed, in 1889, Gouy [55,56,57,58], and in 1905, Stodola [59] independently pointed out that the exergy lost in a process is related to the environmental temperature and to the entropy variation due to irreversibility [27]. Their results represent improvements in the analysis of irreversibility, which begun when Clausius [12] introduced the thermodynamic quantity entropy to take into account the effect of irreversibility in thermodynamic processes [14,27]. Recently, Gujrati [60,61] generalized the classical quantities of heat and work in order to include their time-dependent irreversible components and obtained their expressions in terms of the instantaneous internal temperature and pressure. These results represent a fundamental basis for the formulation of nonequilibrium thermodynamics because the first law turns into the Gibbs relation and the Clausius inequality was generalized. In this way, the Gouy–Stodola theorem maintains its fundamental meaning in nonequilibrium thermodynamics due to the possibility of considering lost work related to entropy production, confirmed by the Clausius inequality.

Now, we can point out that the entropy production, previously defined by a statistical thermodynamic approach, is simply the entropy generation introduced by the Gouy–Stodola theorem, as clearly summarized in Reference [62].

In summary, from a theoretical viewpoint, the definition of temperature requires the support of [63]

- the zeroth law of thermodynamics, in order to introduce the empirical temperature;
- the second law of thermodynamics, in order to quantitatively define the meaning of hot and cold via the introduction of the concept of absolute temperature and the direction of heat flow.

However, in nonequilibrium thermodynamics, some difficulties emerge in relation to these two laws. Indeed, only the definition and the meaning of absolute temperature and entropy cannot be easily defined in conditions out of equilibrium [64] due to the difficulty in identifying the macroscopic variables useful for describing the nonequilibrium states. In order to avoid this difficulty, irreversible thermodynamics introduced the local equilibrium hypothesis [41,63,65,66,67]. In accordance with this hypothesis, the fundamental thermodynamic quantities do not require new definitions, but the two previous laws maintain, locally, their validity. On the contrary, when it is not possible to accept the local equilibrium hypothesis, absolute temperature and entropy must be defined from a new viewpoint [68,69,70,71,72]. These topics are largely discussed both theoretically and experimentally in recent papers [73,74,75,76,77]: we refer to this bibliography for further improvements. Moreover, local equilibrium has been developed in extended irreversible thermodynamics [78,79,80,81], but recently, Grmela et al. [82,83] developed a fundamental approach only based on the dissipation potential, which is a quantity closely related to entropy production.

In this paper, starting from all of these results, we develop an approach to temperature based on the Gouy–Stodola theorem in order to take into account irreversibility and to suggest an approach useful in applied physics and engineering.

## 2. Materials and Methods

During the 13th century, St. Thomas Aquinas (1225–1274) stated that it is impossible for an effect to be stronger than its cause [84]. This implicit statement on irreversibility shows how the effects of irreversibility have always been present in human activities. In the history of the study of irreversibility, a fundamental step is represented by the results of Lazare Carnot [32] on the conservation of mechanical energy for mechanical systems, which today, in thermodynamics, we call closed systems. Indeed, considering any system together with its environment, the total energy is a conserved quantity. The energy lost by the system due to irreversibility flows into the system environment, and it can be evaluated by the Gouy–Stodola theorem [27,36,55,56,58,59,85]:

(6) $$W_{\lambda }=T_{0}\;\Sigma \geq 0$$

where $W_{\lambda }$ is the energy lost due to irreversibility; $T_{0}$ is the environmental temperature; and Σ is the entropy production (Equation (5)), named also entropy generation [27]. Indeed, Equation (5)) points out that, in steady-state systems, the heat wasted from the system to the environment is more probable than the heat absorbed by the system in the same conditions.

Following Garden et al., we consider the system in a nonequilibrium state as a thermal system composed of two different parts [86]:

- One part is composed of the usual classical interaction with the environment, with a heat capacity $C_{eq}$ and the usual equilibrium temperature $T_{eq}$.
- Another part is composed by a continuous energy fluctuation, with the heat capacity $C_{neq}$ and the effective temperature $T_{neq}$.

The second part of the system continuously exchanges heat due to its nonequilibrium, generating the entropy production Σ, as Garden et al. proved by using the Onsager and Prigogine approach. Consequently, nonequilibrium steady-state systems always dissipate heat [87]. Consequently, we introduce the following expression:

(7) $$\Sigma =k_{B}\;\ln\left(\frac{e^{-\left(E_{neq}-\langle E\rangle \right)/\left(k_{B}\;T_{neq}\right)}}{e^{\left(E_{neq}-\langle E\rangle \right)/\left(k_{B}\;T_{neq}\right)}}\right)$$

where $E_{neq}$ is the local energy at the nonequilibrium state and 〈E〉 is its mean value, globally evaluated on the whole system considered. Therefore, we can introduce the explicit definition of nonequilibrium temperature as follows:

(8) $$T_{neq}=2\;\frac{E_{neq}-\langle E\rangle }{\Sigma }$$

In relation to this result, we wish to introduce some comments. Indeed, in order to develop our analysis on the nonequilibrium temperature, we consider the approach introduced by Tool on *fictive temperature*, a parameter useful in conciliate theory and experiments on the glass transition [88,89]: the fictive temperature has the same physical dimension as a temperature, and it describes how far a system is from its initial equilibrium state. This quantity is used both in theoretical and experimental analysis of the vitreous state or on systems with slow internal dynamics [90]. Then, Nieuwenhuizen developed a new thermodynamics, starting from the concept of the fictive temperature [91], obtaining the solution for many open problems related to glass transition [92]. Even if the physical meaning of this temperature is still under discussion from a statistical mechanics viewpoint, we can highlight that, for any system undergoing an irreversible transformation, the fictive temperature has been proven, in a very general way, to be the temperature for systems out of thermodynamic equilibrium for all systems departed from equilibrium generating a positive amount of entropy [86]. Consequently, following the approach fully developed and detailed in Reference [86], the temperature of nonequilibrium defined in (8) can be introduced as a fictive temperature for any state out of equilibrium, locally different from the environmental temperature, and results in a parameter that does not require the notion of local equilibrium to be defined and that represents the nonequilibrium temperature for a nonequilibrium state.

Now, we must link this result to physical measurable quantities. To do so, we introduce the Gouy–Stodola theorem (6), obtaining the following:

(9) $$T_{neq}=2T_{0}\;\frac{E_{neq}-\langle E\rangle }{W_{\lambda }}$$

which, at the equilibrium, becomes

(10) $$T_{eq}=2T_{0}\;\frac{\langle E_{neq}-\langle E\rangle \rangle }{W_{\lambda }}=T_{0}$$

due to equilibrium $\langle E_{neq}-\langle E\rangle \rangle =W_{\lambda }/2$ [11,63], in accordance with the local equilibrium and the zeroth law [2,63]. Moreover, this last relation could lose its meaning if $E_{neq}=\langle E\rangle$ because Σ=0; however, in this case, $W_{\lambda }=0$ due to the previous relation $\langle E_{neq}-\langle E\rangle \rangle =W_{\lambda }/2$ [11,63] and

(11) $$T_{eq}=\lim_{E_{neq}\to \langle E\rangle }2T_{0}\;\frac{\langle E_{neq}-\langle E\rangle \rangle }{W_{\lambda }}=T_{0}.$$

From the previous analysis, $W_{\lambda }$ is the energy wasted towards the environment as a consequence of irreversibility. It is possible to evaluate this quantity using a general approach by relating it to the power of the electromagnetic wave [93]:

(12) $$\begin{array}{c} \dot{W_{\lambda }}=\frac{W_{\lambda }}{\tau }=N\;\left(\frac{1}{2}\epsilon_{0}cE_{el}^{2}+\frac{1}{2\mu_{0}}cB_{m}^{2}\right)\;A \end{array}$$

where τ is the relaxation time, *N* is the number of photons of the electromagnetic wave emitted due to heat generated by irreversibility, $E_{el}$ (V m${}^{-1}$) is the electric field, $B_{m}$ (T) is the magnetic field, *c* is the velocity of light, $\epsilon_{0}=8.854\times 10^{-12}$ (N m${}^{2}$ A${}^{-2}$ s${}^{-2}$) is the electric permittivity in a vacuum, $\mu_{0}=4\pi \times 10^{-7}$ (H m${}^{-1}$) is the magnetic permeability in a vacuum, and *A* is the area (m${}^{2}$) of the interaction surface considered. Therefore, Equation (9) is formulated:

(13) $$T_{neq}=2\frac{\tau }{A}\;\frac{\Gamma }{\frac{1}{2}\epsilon_{0}cE_{el}^{2}+\frac{1}{2\mu_{0}}cB_{m}^{2}}\;T_{0}$$

where $\Gamma =(E_{neq}-\langle E\rangle )/N$ is the mean energy density due to the difference between the nonequilibrium value of energy at $T_{neq}$ and its mean value at the same temperature [94]. This result points out that the nonequilibrium temperature is proportional to the characteristic time of the system. Moreover, the electronic noise is due to the thermal motion of the electrons inside the matter and, thus, the measure of the frequency of the noise represents an indirect measure of the nonequilibrium temperature because the noise is generated by the collisions of the electrons at the frequency ν=1/τ.

## 3. Results

In this paper, we improved our previous analyses of the nonequilibrium temperature.

The first result is to obtain a definition of nonequilibrium temperature related to the environmental temperature. This result is in accordance with the main theoretical and experimental results in the literature; indeed, all the results in the literature point out that the nonequilibrium state is cause by an interaction with the system environment [4], and in Equation (13), the nonequilibrium temperature is directly proportional to the environmental temperature.

The second result is to link the nonequilibrium temperature to the wasted energy expressed by the electromagnetic power of the photons outflow, related to the interaction among the internal structures of the system considered: electron–atoms (or molecules), electron–electron, atoms–atoms (or molecules–molecules), electrons–lattice, etc.

The third result is to introduce the relaxation time τ, related to the system response to any perturbation/fluctuation. Moreover, in this approach, the local equilibrium is not required to obtain the result (13).

Lastly, the result highlights the characteristic quantity of all of the thermal fluxes and the area of interaction, pointing out the fundamental role area of interaction of any system. Moreover, all of the physical quantities in the relation can be directly measured or evaluated by classical or quantum physics.

Now, in order to show an application, we theoretically evaluate the result obtained for a solid without any other perturbation (electromagnetic, electric, mechanic, etc.) except the spontaneous thermal interaction between the system and its environment. Considering a solid of volume *V* and length *L*, the value of Γ for a solid band with energy *E* can be evaluated using the free-electron model of a solid [94], obtaining the following:

(14) $$\Gamma =\frac{h^{3}}{8\pi \sqrt{2}\;m_{e}^{3/2}}\;\frac{1}{\sqrt{E}}$$

where $h=6.626\times 10^{-34}$ J s is the Planck constant and $m_{e}\approx 9.11\times 10^{-31}$ kg is the mass of the electron. If we consider $E=E_{max}$, with $E_{max}=h^{2}/8m_{e}a$ being the maximum energy in the band and *a* being the distance between two lattice knots, we can highlight that this quantity is independent from the number of electrons in the band and we can obtain our last result for the definition of nonequilibrium temperature in a solid in the free-electron model approximation:

(15) $$T_{neq}=2\frac{T_{0}\;L}{V}\;\frac{\frac{h^{2}a^{1/2}}{4\pi m_{e}}}{\frac{1}{2}\epsilon_{0}cE_{el}^{2}+\frac{1}{2\mu_{0}}cB_{m}^{2}}\;\tau =\frac{\mu_{0}\;h^{2}a^{1/2}}{\pi m_{e}}\;\frac{\tau }{\sqrt{\epsilon_{0}\mu_{0}}E_{el}^{2}+cB_{m}^{2}}\;\frac{L}{V}\;T_{0}$$

where we considered that V/L≈A.

The relaxation time τ can be obtained by using the Drude–Lorentz theory [94]:

(16) $$\tau =\frac{m_{e}\sigma_{R}}{ne^{2}}$$

where $\sigma_{R}$ is the conductivity $\Omega^{-1}$ m${}^{-1}$ and *n* is the number of electrons per unit volume. Consequently, the nonequilibrium temperature is as follows:

(17) $$T_{neq}=\frac{\mu_{0}\;h^{2}a^{1/2}}{n\pi e^{2}}\;\frac{\sigma_{R}}{\sqrt{\epsilon_{0}\mu_{0}}E_{el}^{2}+cB_{m}^{2}}\;\frac{L}{V}\;T_{0}$$

The link between nonequilibrium temperature and the characteristic time imply that nonequilibrium temperature is related to the universal computation and the ubiquitous 1/ν phenomena [95,96]. Therefore, we agree with the results of Chua et al., who proved that there exists a fundamental relationship between universal computation and the ubiquitous 1/ν phenomena [97]. In this context, the results obtained by Yadati et al. represent numerical and experimental proofs of our results [73] in relation to their experiments and statistical analysis, able to quantify spatiotemporal thermal fluctuations in a driven out-of-equilibrium steady-state system, with particular interest in the convection patterns of a Rayleigh-Bènard fluid cell at steady-state.

## 4. Discussion and Conclusions

Interest in nonequilibrium temperature is growing due to its fundamental role in recent improvements in and applications of nonequilibrium thermodynamics, information theory, kinetic theory, nonequilibrium molecular dynamics, granular fluids, supercooled liquids under shear, radiative systems, or fundamental grounds [98].

All of the studies on nonequilibrium temperature have emphasized that, out of equilibrium, the definition of temperature must be related to different aspects of the system [99]. Moreover, nonequilibrium temperature has been shown to depend both on the energy of the system and on the energy flux [98] between the system and its environment, which are related to the power of the forces, which maintain the oscillator, and to the viscous forces [99].

It has been pointed out that nonequilibrium thermodynamics could be related to the average energy and to the fluxes acting on the system [63].

In this paper, we introduced a definition of temperature that satisfied all of the theoretical requirements. Indeed, in our definition of nonequilibrium temperature, we have linked it to the following:

- The electromagnetic outflow, generated by irreversibility during microscopic interaction in the system, as a consequence of the Carnot approach to systems and the Gouy–Stodola theorem;
- The environmental temperature;
- The mean energy of the system;
- The relaxation time τ and the geometrical and physical characteristics of the system.

Now, we consider that it could be useful to suggest an example of use of the results obtained. Transport phenomena are fundamental processes in systems out of equilibrium. Mass transfer can be obtained by the application of directional force on the mass. When a selected portion of a bistable potential is subjected to heat exchange, the relative stability of the two wells differs as a result of which some proportion of mass is transferred from the perturbed well to the other: this phenomenon is known as Landauer blowtorch effect [100]. In this effect, the temperature difference represents the cause of the mass transfer; indeed, the extra kinetic energy gained by the particles at the high temperature region is able to supply the energy required to cross a potential barrier. To analyze this effect, we consider *N* identical Brownian particles of mass *m* in a bistable completely symmetric potential V(x) such that V(−x)=V(x)), where *x* is a position variable. This potential presents a maximum in $x=x_{max}=0$ and two minima, in $x=-x_{min}$ and in $x=x_{min}$, respectively. The temperature of the system is *T*. Heat exchange occurs at temperature $T_{neq}$ only in the positions range $x\in [-x_{1},-x_{2}]$, such that $-x_{min}\leq -x_{1}\leq -x_{2}\leq 0$. Therefore, the maximum represents the potential barrier. The heat transfer results as follows:

(18) $$Q=\int_{-x_{1}}^{-x_{2}}V\left(x\right)dx$$

and the entropy variation is as follows [101]:

(19) $$\Sigma =\Delta S=\left(\frac{1}{T_{neq}}-\frac{1}{T}\right)\left[V\left(-x_{2}\right)-V\left(-x_{1}\right)\right]$$

where *S* is the entropy. Consequently, the two relative population densities in steady state are changed as follows [101]:

(20) $$\frac{P_{R}}{P_{L}}=\frac{P_{R,eq}}{P_{L,eq}}exp\left\{\left(\frac{1}{T_{neq}}-\frac{1}{T}\right)\left[V\left(-x_{2}\right)-V\left(-x_{1}\right)\right]\right\}$$

where *P* is the integrated probability of residence of the particle in the state *L* or *R*, and where *L*, *R*, and eq mean left, right, and equilibrium, respectively. It is possible to highlight that the entropy increases as a consequence of flows of Brownian particles across the barrier, with a variation of the relative population density in the two wells [100]. Then, we consider the following:

(21) $$\frac{P_{R}}{P_{L}}-\frac{P_{R,eq}}{P_{L,eq}}=\frac{P_{R,eq}}{P_{L,eq}}\left(exp\left\{\left(\frac{1}{T_{neq}}-\frac{1}{T}\right)\left[V\left(-x_{2}\right)-V\left(-x_{1}\right)\right]\right\}-1\right)$$

and the flow of mass can be obtained as follows [101]:

(22) $$\dot{m}=m\int_{-x_{1}}^{-x_{2}}n\frac{P_{R,eq}}{P_{L,eq}}\left(exp\left\{\left(\frac{1}{T_{neq}}-\frac{1}{T}\right)\left[V\left(-x_{2}\right)-V\left(-x_{1}\right)\right]\right\}-1\right)dx$$

where *n* is the linear density of particles. Now, if $|T_{neq}-T|<<T$, then the mass flows becomes [101]:

(23) $$\dot{m}=m\frac{P_{R,eq}}{P_{L,eq}}\left[\frac{T_{neq}-T}{T^{2}}\left(V\left(-x_{2}\right)-V\left(-x_{1}\right)\right)\right]\int_{-x_{1}}^{-x_{2}}ndx$$

This approach allows us to explain the macroscopic effect of the diffusion in a temperature gradient, named Soret effect, which is still under analysis for better comprehension. Indeed, the Soret effect is important in civil engineering due to its relation to water motion in presence of thermal bridges [101]. Therefore, we highlight it as our approach represents a new viewpoint for engineering and physical applications.
